# Health information-seeking behavior associated with linguistic group membership: latent class analysis of a population-based cross-sectional survey in Italy, August to September 2014

**DOI:** 10.1186/s13690-022-00847-w

**Published:** 2022-03-21

**Authors:** Dietmar Ausserhofer, Wolfgang Wiedermann, Ulrich Becker, Anna Vögele, Giuliano Piccoliori, Christian J. Wiedermann, Adolf Engl

**Affiliations:** 1 Institute of General Practice, College of Healthcare Professions Claudiana, Lorenz-Böhler-Street 13, 39100 Bolzano, Italy; 2 Institute of Nursing Science, Department Public Health, University of Basel, Basel, Switzerland; 3 Department of Educational, School and Counseling Psychology, College of Education and Human Development, Missouri Prevention Science Institute, University of Missouri, Columbia, MO USA; 4 Apollis Institute of Social Research and Opinion Polling, Bolzano, Italy

**Keywords:** Linguistic ethnicity, Health information-seeking behavior, Population-based survey, Latent class analysis

## Abstract

**Background:**

Evidence suggests an increasing demand for culturally and linguistically responsive disease prevention programs and health interventions. It is important to understand how individuals seek health information to address the potential needs of the health care system.

**Methods:**

Latent classes of health information-seeking behaviors in a linguistically mixed region of Italy were explored through a population-based telephone survey of ten health information sources. Data were collected in August and September 2014 from 504 adults in South Tyrol, Italy (primary language German, 68%; Italian, 28%), and analyzed using latent class analysis and latent class multinomial logistic regression models.

**Results:**

Three classes of health information-seeking behaviors emerged: “multidimensional” (23.3%), “interpersonal” (38.6%) and “technical/online” (38.1%). Compared to the “technical/online” class, “interpersonal” class members were older, had lower education than high school, and were less likely to be of Italian ethnicity. “Multidimensional” class members were more likely to be female, older, and of German ethnicity than those in the “technical/online” class.

**Conclusions:**

Linguistic ethnicity explains membership in classes on health information-seeking behavior. Policymakers and healthcare providers should consider the health information-seeking behaviors of population subgroups to promote health and medical care in linguistic minority groups.

**Supplementary Information:**

The online version contains supplementary material available at 10.1186/s13690-022-00847-w.

## Background

Health information-seeking behavior (HISB) refers to a strategy whereby people develop a social and personal sense of health to understand their health problems, reduce uncertainty regarding health status, and cope with illness [1]. Utilizing recent developments in technologies and networks, people increasingly seek health information on the Internet, particularly when it comes to receiving conflicting health information or when the cause is relevant to family members and friends [2]. Studies on the characteristics of individuals’ motives for engaging in HISB document the types of health and medical information being sought not only in the context of disease and illness but also in healthy individuals pursuing information to maximize positive health outcomes [3]. HISB analysis can be incorporated into studies to understand how various communities seek information in online versus non-online contexts [4].

Although there is strong international evidence that language barriers present obstacles to healthcare access, quality, and safety, little research has been conducted on the HISB of official language minorities [5]. It is important to understand how individuals seek health information to address potential deficits in healthcare systems in medical care. Evidence suggests an increasing demand for disease prevention programs and health interventions that are more culturally and linguistically responsive [6–8]. Language plays a critical role in HISB, as demonstrated in studies of linguistic minority groups [9, 10]. Nativity and language preferences are significant determinants of seeking health information [11]. In addition to cultural characteristics and language preferences, online tool ownership and use are important predictors of how health information is sought [12].

Italy adopted a mandatory vaccination policy in 2014 to achieve target immunization coverage rates and to experience outbreaks. In July 2017, the Italian Ministry of Health approved law N 119, which has extended the number of mandatory vaccinations for school attendance from four to ten. In particular, vaccination against poliomyelitis, diphtheria, tetanus, pertussis, hepatitis B, Hib disease, measles, mumps, rubella, and varicella has become mandatory for kindergarten attendance [13]. Vaccine hesitancy for mandatory vaccinations is higher in Trentino – Alto Adige than in other regions of Italy [14], and vaccine uptake in South Tyrol has remained below the Italian average for years, for example, for measles at 77 per cent vs. 92 per cent (year 2020; 2018 cohort at 24 months of age) [15]. South Tyrol, the Autonomous Province of Bolzano, is part of the Trentino–Alto Adige region in Italy, next to Austria (total population, 524.256), with approximately 70 percent German and 25 percent Italian-speaking inhabitants [16]. Different linguistic groups are served by the same healthcare system and service provider.

Considering the overall positive effect of the introduction of child vaccine mandates [17, 18], Italy extrapolated this policy to COVID-19 vaccination [19]. In the coronavirus pandemic, weekly incidence rates put South Tyrol among the worst of all Italian regions, and the vaccination rate is the lowest in Italy, although public health care quality is considered above average [20]. While the majority in South Tyrol are German speakers, Italian speakers tended to be more obedient during the coronavirus pandemic. Correspondingly, 20 percent of the school staff at German language schools were unvaccinated compared to 3 percent in Italian schools [21]. Given the historic disinclination to accept orders from the central government, German speakers in South Tyrol seem to have lower levels of trust in the health authorities. A survey in May 2021 found that 44 percent of German speakers trusted the Italian National Institute of Health recommendations, compared to 70 percent of Italian speakers [21]. South Tyrol had low levels of childhood vaccination before COVID, with instead of the range of 90 percent only 71.9 percent of children 24 months of age jabbed against measles in 2017 [22].

To improve the potential deficits in health literacy, prevention, and medical care, it is helpful to better understand the linguistic characteristics of HISBs. The main objective of this study was to assess and describe HISB in the South Tyrolean population. The aims were to (1) explore latent classes of HISB and (2) test the association between socio-demographic characteristics (i.e., age, gender, educational level, region of origin, and linguistic group) and latent class membership.

## Methods

### Sample and procedure

Data were derived from a population-based cross-sectional telephone survey that was initiated before the coronavirus pandemic because of the historically low vaccination rates in South Tyrol. The eligibility criteria for participants were living in South Tyrol, possessing a landline (only private households, no business phones), being at least 18 years old, and being declared to either the German or the Italian linguistic group. According to data from the National Statistics Institute (www.dati.istat.it) for 2015, from a total of about 210,000 private households in South Tyrol, 122,000 (58%) had a landline, 208,000 (99%) of private households had at least one mobile phone, and 92,000 (44%) used mobile phones. In the present study, only landline users were included, because mobile phone users cannot be limited to South Tyrol. Computer-assisted telephone interviews were conducted between August and September 2014. Data collection was conducted by Apollis (www.apollis.it), a private research institution in Bolzano (BZ), Italy, conducting empirical studies for public and private clients with a focus on education, labor market topics, active aging, and survey research. The goal was to conduct at least 500 interviews. Professional interviewers contacted a random sample of 1,445 households in South Tyrol with landline numbers. A total of 162 phone numbers were incorrect and 318 were not reachable. The remaining 965 were invited to participate in the telephone survey and appointments were made to answer the phone survey: 458 did not participate in the second call despite the appointment, for 46 no suitable appointment was found during the study period, 53 were not capable of participating, and 359 declined. Of the 507 phone interviews, three were excluded from the analyses as participants were not eligible, resulting in a total of 504 interviews. The interviews lasted from three to 28 min (mean,12 min).

### Measurements

We assessed ten common sources of health-related information: (1) medical layperson discussion (being asked by others for advice), (2) magazines/newspapers, (3) TV/radio, (4) friends, (5) healthcare professionals, (6) courses, (7) medical literature, (8) random online search, (9) targeted online search in electronic databases, and (10) online forums. These items were self-developed based on existing instruments without validation and assessed on a 4-point Likert scale (ranging from 1 = never to 4 = often). Participants’sociodemographic factors included age (birth year), sex (male/female), mother’s language (German/Italian), educational level (highest degree), and region of origin (rural/urban). The survey items and dimensions are listed in the Appendix (see additional files 1 and 2).

### Statistical analysis

We used descriptive statistics, including means, standard deviations, frequencies, cross-tabulations to describe the HISBs, and the characteristics of the sample. To avoid bias, sampling weights based on the age and sex distributions for the population of South Tyrol according to the Provincial Statistics Institute for 2013 were employed for all analyses [16].

Evaluating health information patterns using the original ordinal responses would have required larger sample sizes. Therefore, for the purpose of the present analysis, health information items of the survey were dichotomized with 0 = “never”/”rarely” and 1 = “sometimes”/ “often.” The response categories “never” and “rarely” were collapsed to avoid sparse cell frequencies in subsequent analyses. According to our research aims, we analyzed the data in two steps. First, we completed a latent class analysis (LCA) to explore whether meaningful latent classes of people’s HISB could be identified from the 10 dichotomous health information indicators. LCA is a statistical model used to identify the underlying mutually exclusive and exhaustive subgroups of individuals with shared characteristics [23]. Because the number of latent classes is unknown a priori, a series of LC models with one–five latent classes were estimated. To avoid local maxima of log-likelihoods, 1000 random starts were used for each model. To select the appropriate number of classes, Akaike information criterion (AIC) and Bayes information criterion (BIC) were applied (lower values indicate a better model fit). In addition, unadjusted and adjusted Lo-Mendell-Rubin (LMR) tests were used to evaluate whether the *k*-class solution was superior to the *k* – 1 class solution. A significant LMR test suggests that the *k*-class solution fits the data better than the *k* – 1 class solution. In addition to the statistical indices, the interpretability of the model coefficients was inspected for each model [24]. LCA assumes that the underlying latent classes explain why the observed indicators are related to each other (known as the local independence assumption). Standardized bivariate residuals were used to assess potential violations of the local independence assumption. Because linguistic group-specific sampling weights were not available, LC modelling was performed using the total sample.

After latent class enumeration, a latent class multinomial logistic regression model was used to predict the class memberships. Note that a two-step approach (in which modally assigned LC memberships are used as dependent variables in multinomial logistic regression) is prone to overestimate the influence of predictors [25]. Thus, after determining the optimal number of classes, a one-step approach was applied, where class identification and LC membership prediction were performed simultaneously [26] based on the respondents’ age (in years), gender (0 = male, 1 = female), education (0 = less than high school, 1 = high school +), region (0 = urban, 1 = rural), and linguistic group (0 = German, 1 = Italian). The level of significance was set at *p* < 0.05. Data analysis was conducted using Mplus version 7.3 [27]. Only four observations had missing values for health information indicators. Full-information maximum likelihood estimation was applied to handle these missing data points. In addition, 20 participants (4.0%) had missing values for at least one of the covariate. No significant differences in demographics and health-information seeking behavior were observed for this small subgroup, which was discarded from latent-class multinomial logistic regressions, leaving *n* = 484 subjects. When predicting class memberships, the main effects and potential moderation effects of covariates on the relationship between linguistic groups and latent class memberships were investigated [28].

## Results

Data were collected from 504 participants, amounting to a response rate of 52%. The mean age was 48.9 years (SD = 18.7; range: 18 – 98), 49% of the participants were male, and the primary languages were German (68%) and Italian (28%). The respondents’sociodemographic characteristics are summarized in Table 1.

**Table 1:** Descriptive statistics of weighted sample by linguistic group of a population-based cross-sectional survey in Italy, August to September 2014

		Linguistic Group		
Variable		German	Italian	Total
Age (years)	*M* (*SD*)	47.9 (18.7)	51.1 (18.6)	48.9 (18.7)
Sex	*n* (%)			
Male		168 (49.1)	70 (49.6)	238 (49.3)
Female		174 (50.9)	71 (50.4)	245 (50.7)
Educational Level	*n* (%)			
less than high school		210 (61.2)	61 (43.3)	271 (56.0)
high school +		133 (38.8)	80 (56.7)	213 (44.0)
Region	*n* (%)			
urban		80 (23.3)	115 (81.6)	195 (40.3)
rural		263 (76.7)	26 (18.4)	289 (59.7)
Source of Information				
Beeing asked for advice	*n* (%)			
never/rarely		166 (48.5)	66 (46.8)	232 (48.0)
sometimes/often		176 (51.5)	75 (53.2)	251 (52.0)
Newspaper/Magazines	*n* (%)			
never/rarely		105 (30.7)	44 (31.2)	149 (30.8)
sometimes/often		237 (69.3)	97 (68.8)	334 (69.2)
TV/Radio	*n* (%)			
never/rarely		107 (31.2)	63 (44.7)	170 (35.1)
sometimes/often		236 (68.8)	78 (55.3)	314 (64.9)
Friends	*n* (%)			
never/rarely		99 (28.9)	57 (40.1)	156 (32.2)
sometimes/often		244 (71.1)	85 (59.9)	329 (67.8)
Professionals	*n* (%)			
never/rarely		181 (52.9)	59 (42.8)	240 (50.0)
sometimes/often		161 (47.1)	79 (57.2)	240 (50.0)
Courses	*n* (%)			
never/rarely		272 (79.3)	119 (83.8)	391 (80.6)
sometimes/often		71 (20.7)	23 (16.2)	94 (19.4)
Medical Literature	*n* (%)			
never/rarely		227 (66.2)	102 (72.3)	329 (68.0)
sometimes/often		116 (33.8)	39 (27.7)	155 (32.0)
Random Online Search	*n* (%)			
never/rarely		194 (56.6)	68 (47.9)	262 (54.0)
sometimes/often		149 (43.4)	74 (52.1)	223 (46.0)
Targeted Online Search	*n* (%)			
never/rarely		198 (57.7)	87 (61.7)	285 (58.9)
sometimes/often		145 (42.3)	54 (38.3)	199 (41.1)
Online Forums	*n* (%)			
never/rarely		327 (95.6)	131 (92.9)	458 (94.8)
sometimes/often		15 (4.4)	10 (7.1)	25 (5.2)

*n* Frequencies, *M* Mean, *SD* Standard deviation

As different response rates in different groups may lead to selection bias, the data structure was matched with the address database and official register of the population census 2011. Deviations were assessed using a multilevel weighting process and balanced according to address databases of statistical districts and rural/urban weighting, including adaptations, after January 1, 2013, census by language group, gender, and age group. The calculated weights ranged from 0.29 to 3.82, with the highest weights assigned to young women in urban areas. These were examined on a case-by-case basis to rule out possible outliers that could have distorted the results. The random sample was considered representative of the population.

Of the ten HISBs (see Table 1), the three most frequent behaviors were seeking information in “Newspapers/Magazines” (69.2%), from “Friends” (67.8%), and in “Radio/TV” (64.9%). The three less frequent health-information seeking behaviors were “Online Forums” (5.2%), “Courses” (19.4%), and “Medical Literature” (32.0%).

Table 2 summarizes the LC model fit indices and estimated class sizes (based on modal assignment) for *k* = 1 – 5 latent classes. For comparison, the LC models were estimated with and without sampling weights. In general, AIC values decrease with every additional class, which hampers the selection of distinct models. Because this is also in line with the observation that AIC tends to overestimate the number of latent classes, we primarily focused on BIC. For both the weighted and unweighted LC models, the BIC favored the 3-class solution. The adjusted and unadjusted LMR tests also favored the 3-class solution when no sampling weights were used and suggested a 2-class solution when sampling weights were incorporated. Differences in parameter estimates obtained from the weighted and unweighted 3-class models were modest, and the weighted 3-class model showed an acceptable model fit (χ^2^(991) = 973.48, *p* = 0.648). Thus, we retained the weighted 3-class solution as the final model. Note that this model showed one significant standardized bivariate residual involving the health information sources newspapers/magazines and TV/radio, suggesting a significant residual covariance. Because qualitatively different patterns of media use occurred among the extracted latent classes, instead of compiling the two indicators into a global “media” indicator, we kept the indicators separate and accounted for these associations by re-estimating the final model while allowing the corresponding residual covariance.

**Table 2:** Summary of latent class model fit for with and without sampling weights (indices suggesting best model fit are marked bold) of a population-based cross-sectional survey in Italy, August to September 2014

					*Latent class sizes based on modal assignment*				
No. of classes	AIC	BIC	LMR	adj. LMR	LC1	LC2	LC3	LC4	LC5
*Without sampling weights*									
1	5918.6	5960.8	-	-	504	-	-	-	-
2	5656.4	5745.1	< .0001	< .0001	182	322	-	-	-
3	5587.0	**5722.1**	**0.010**	**0.011**	156	107	241	-	-
4	5554.8	5736.4	0.213	0.219	136	111	206	51	-
5	**5539.8**	5767.9	0.603	0.607	85	144	62	64	149
*With sampling weights*									
1	6041.1	6083.3	-	-	504	-	-	-	-
2	5836.2	5924.9	** < .001**	** < .001**	222	282	-	-	-
3	5771.9	**5907.0**	0.203	0.209	197	103	204	-	-
4	5736.3	5917.9	0.749	0.749	127	125	142	110	-
5	**5714.7**	5942.7	0.742	0.743	86	122	58	106	131

*AIC* Akaike information criterion, *BIC* Bayes information criterion, *LMR P*-value of the Lo-Mendel-Rubin test, *adj. LMR p*-value of the adjusted LMR

**Fig. 1:**
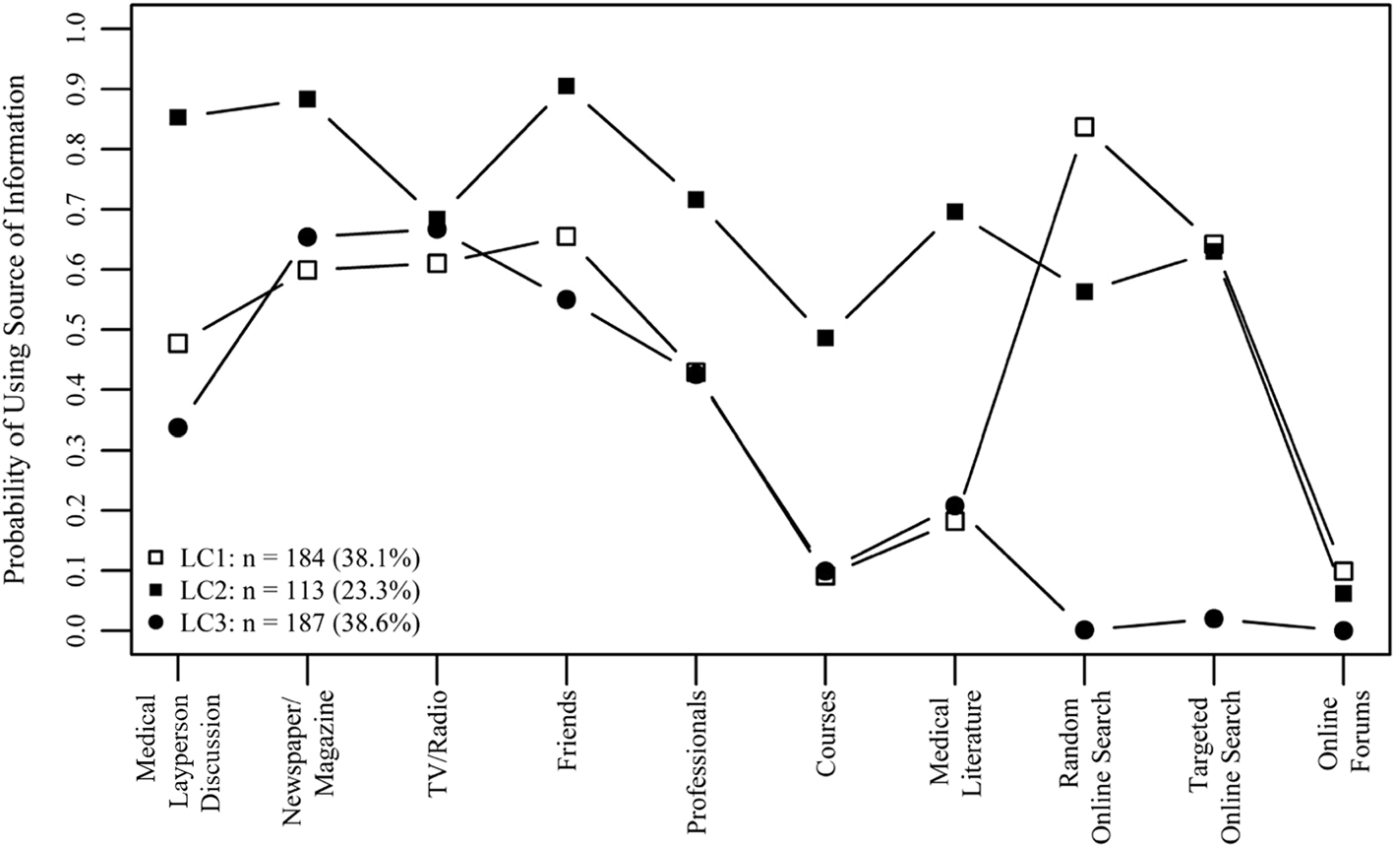
Sources of health information of different latent classes of information-seeking groups of a population-based cross-sectional survey in Italy, August to September 2014. LC1, “technology/online” pattern; LC2, “multidimensional” pattern; LC3, “interpersonal” pattern (based on weighted sample)

LC-specific patterns of health information-seeking behaviors are summarized in Fig. 1, and can be described as “multidimensional,” “interpersonal,” or “technical/online.” Members of the “multidimensional” group (23.3%) reported performing almost all behaviors of seeking health information, ranging from exchanges with friends, health professionals, or other human sources to using medical literature, electronic databases, or the internet. The “interpersonal” group (38.6%) was more likely to seek information from friends or health professionals, while the “technical/online” group (38.1%) was more likely to use random or targeted online searches in electronic databases or the internet.

Next, the covariates were entered into the LC model to predict the latent class memberships. The descriptive characteristics of the three latent classes are summarized in Table 3.

**Table 3:** Descriptive statistics of weighted sample by latent class membership of a population-based cross-sectional survey in Italy, August to September 2014

		Latent Class Membership		
Variable		LC1: "technical/online"	LC2: "multidimensional"	LC3: "interpersonal"
Class Size	*n* (%)	184 (38.1)	113 (23.3)	187 (38.6)
Linguistic group	*n* (%)			
German		110 (32.1)	95 (27.7)	138 (40.2)
Italian		75 (53.2)	18 (12.8)	48 (34.0)
Sex	*n* (%)			
Male		109 (45.6)	31 (13.0)	99 (41.4)
Female		76 (31.0)	81 (33.1)	88 (35.9)
Educational Level	*n* (%)			
less than high school		76 (28.0)	40 (14.8)	155 (57.2)
highschool +		108 (50.7)	73 (34.3)	32 (15.0)
Region	*n* (%)			
rural		88 (45.1)	45 (23.1)	62 (31.8)
urban		96 (33.2)	68 (23.5)	125 (43.3)
Age (years)	*M* (*SD*)	35.8 (14.6)	46.9 (13.2)	62.9 (15.0)

*M* Mean, *SD* Standard deviation

In addition to testing the main effects of class membership, all potential two-way interactions were investigated. Non-significant predictors were omitted from the multinomial logistic regression models because of their parsimony. In general, no significant differences were observed between urban and rural regions. Therefore, this covariate is excluded from the model. Similarly, none of the two-way interactions considered were significant. The main effects of the final latent-class multinomial logistic regression are summarized in Table 4.

**Table 4:** Latent class multinomial logistic regression of patterns of health information-seeking behaviors based on weighted sample of a population-based cross-sectional survey in Italy, August to September 2014

	LC2: "multidimensional"			LC3: "interpersonal"		
		95% CI			95% CI	
Variables	OR	lower	upper	OR	lower	upper
Sex: Female	3.41	1.06	10.97	0.90	0.26	3.09
Age (in years)^a^	1.06	1.02	1.10	1.13	1.09	1.18
Linguistic group: Italian	0.18	0.05	0.70	0.27	0.09	0.84
Educational Level: High school +	1.75	0.58	5.25	0.21	0.08	0.60

Reference Class: LC1: "technical/online"

^a^mean centered

**Fig. 2:**
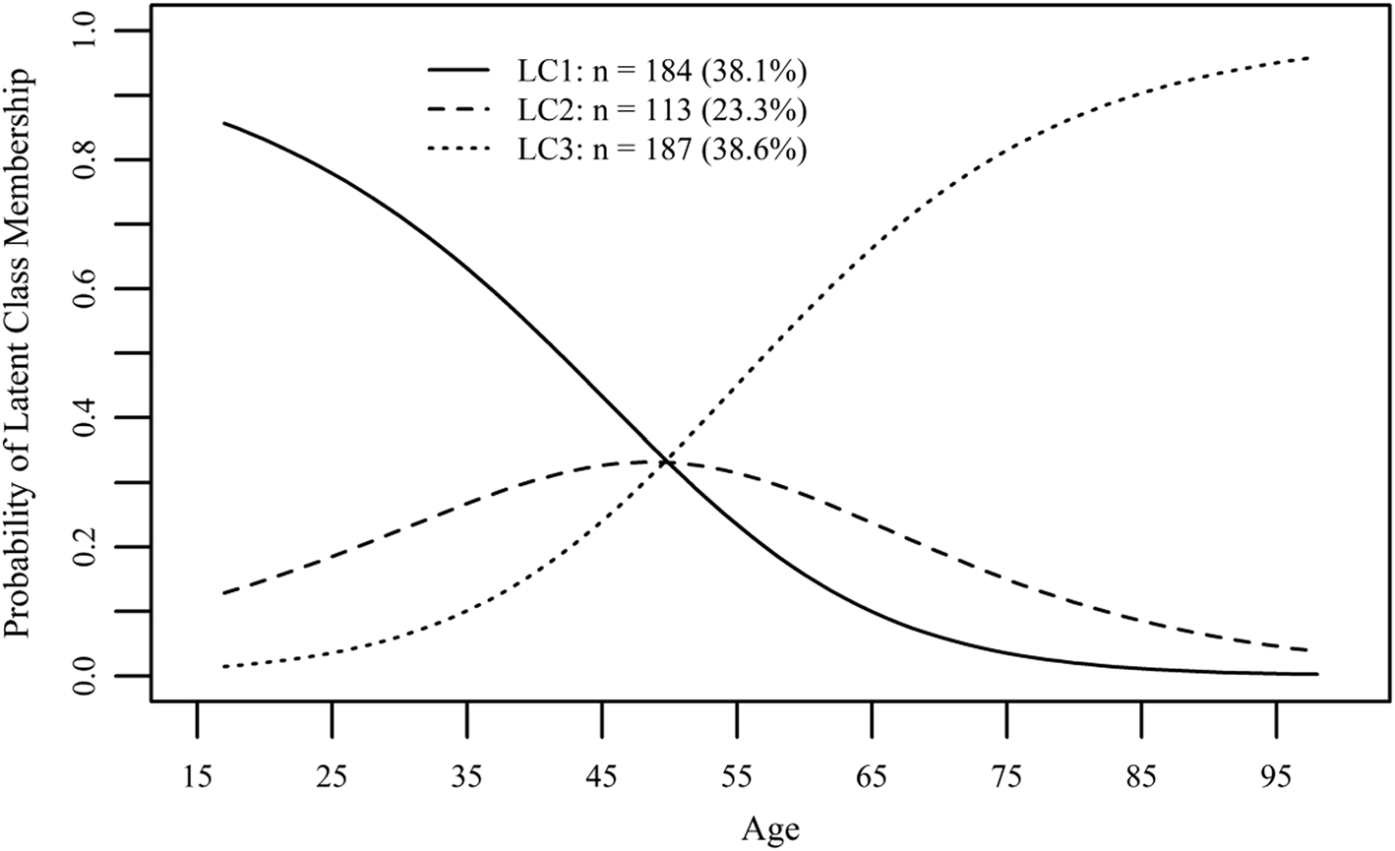
Age distribution of three different latent class analysis health information-seeking groups of a population-based cross-sectional survey in Italy, August to September 2014. LC1, “technology/online” pattern; LC2, “multidimensional” pattern; LC3, “interpersonal” pattern (based on weighted sample)

The upper panel of Table 4 shows the results when using the “technical/online” class as the reference group; the lower panel gives the results when using the “multidimensional” class as the reference group. Compared to the “technical/online” group, members of the “multidimensional” group were significantly more likely to be female, older, and of German ethnicity. No difference was found in educational level. Members from the “interpersonal” group were significantly more likely to have higher age and education lower than high school, and less likely to be of Italian ethnicity than those in the “technical/online” group (upper panel of Table 4). Further, compared to members classified being “multidimensional”, members of the “technical/online” group were less likely to be female and of older age and were more likely to be of Italian ethnicity (the latter effect, however, must be interpreted with caution due to a rather wide 95% CI); members of the “interpersonal” group were less likely to be female and were more likely to have higher age and education lower than high school. In the latter comparison, no significant differences were found across the linguistic groups. As displayed in Fig. 2, the probability of membership in the “technology/online” group decreases with age, whereby the probability of being a member in the “interpersonal” group increases with age. The probability of membership in the “multidimensional” group peaks around 50 years of age and decreases with older age.

## Discussion

The HISB is affected by linguistic variables and is likely to be attributed to the effectiveness of preventive and medical care. In this study, patterns of HISB in the adult population of South Tyrol were investigated for the first time and revealed three health information-seeking groups in LCA, namely “interpersonal,” “technical/online,” and “multidimensional.” While the “multidimensional” group of adults performed almost all behaviors of seeking health information, ranging from exchange with friends, health professionals or other human sources to use of medical literature, electronic data bases and internet, the “interpersonal” group sought information mainly from friends and/or health professionals. The “technical/online” group used random or targeted online search in electronic data bases or the internet. Class membership was explained by age, sex, level of education, and most importantly, ethnicity. HISBs differed significantly between the two linguistic groups.

In regions with culturally and linguistically diverse backgrounds, differences in people’s behaviors when searching for health-related information have been described in the literature [29–31]. A characteristic of the South Tyrolean society is its relatively strong ethnicization. This is primarily reflected in the institutionalized separation by language groups (e.g. linguistically separate school systems, allocation of public service jobs according to "ethnic proportionality"), but can also be clearly demonstrated in the economic sphere (agriculture and tourism as traditionally "German" domains, public service and industry for a long time as the primary economic base of the "Italians"). This ethnicization at its core is based on the economic differences of interest between the language milieus [32]. Being part of the German-speaking minority in an Italian national healthcare system may impact HISB; for instance, drug package inserts are provided in the Italian language only. In contrast to the non-medical public services established in South Tyrol for both German and Italian ethnic groups, there is a single public healthcare system for the entire population. Although the language skills of the German-speaking minority in Italian were not assessed in this study, only a portion of the population is known to be sufficiently bilingual.

Patterns related to the access and usage of digital technologies characterize HISB and affect health literacy [33, 34]. Reliance on Internet-based technologies to disseminate health information and services is well known and has most recently been exemplified during the coronavirus pandemic [35–37]. In Germany, middle and high socioeconomic status, female sex, being married or living in a stable relationship, and heavy use of healthcare services favor the use of the Internet for health-related information [38]. One-third of adults in the general South Tyrolean population primarily seek health information through random or targeted online searches in electronic databases or the internet. This percentage may have increased due to the age of the participants in the dataset. Our study revealed that Italian speaking, male sex, lower age (< 50 years), and higher education increased the likelihood of applying this HISB. As online information offers great potential to empower the population [39], efforts made in the South Tyrol healthcare system to improve access to online health information (e.g., eHealth services) may focus on individuals from the “interpersonal” group to enhance their capacity to use it effectively through educational programs. As described in "Regional Development Strategies 2014–2020" adopted by the South Tyrolean government in 2013 [40], agriculture and tourism will derive significant benefits from e-commerce and e-tourism. The increased use of information and communication technologies creates opportunities for social and economic development in rural areas. The implementation of European and national “Digitization Strategies 2014–2020” at the regional level [41] could have mitigated the linguistic differences in online HISB described here. Although this development is still ongoing, it may be time to repeat the present study because of its relevance to public health.

Access to “interpersonal” health information is at risk, as there here is a particular shortage in German-speaking health care professionals in South Tyrol, which has become a general healthcare problem in Italy today [42]. In our study, friends and health care professionals were two particularly important sources of health-related information for the German-speaking population in the “interpersonal” and the “multidimensional” LCA groups. Moreover, health professionals often have a limited understanding of health literacy levels, including HISB, and the consequences of low health literacy on their patients [43].

German-language inhabitants may be influenced by health information from German-speaking Switzerland, Germany, and Austria because public television in South Tyrol includes public television channels in Switzerland, Germany, and Austria (SRF 1, SRF 2, ARD, ZDF, ORF 1, ORF 2, 3SAT). Regional evening news of ORF 1 and ORF 2 include “Südtirol heute”. When it comes to television news, “Südtirol heute” is among the top ranked in South Tyrol with more than 100,000 viewers a day, which corresponds to 20 percent of the population [44]. Providing health-related information to adults in the “interpersonal” and “multidimensional” groups is language dependent. Healthcare professionals need to assess health literacy levels as well as effectively communicate and consider health literacy among other patient characteristics when selecting patients for care management programs [34]. Health policymakers and healthcare organizations should implement interventions not only to develop health information-seeking skills in the populations they serve, but also to prepare healthcare professionals, such as general practitioners and family nurses, for better provision of information and materials that are easily accessible and understandable [34]. Critical pedagogy applied to in-service education has been shown to effectively stimulate professionals' awareness of their potential to change their practice and work environment towards improved health literacy in special linguistic contexts [18].

LCA has been found useful when considering multiple sources to study HISB, particularly when the capacity to seek multiple sources is unequal [45]. LCA findings provide implications for interventions with respect to subgroups to be prioritized and areas to be targeted in efforts to promote access to and acquisition of health information and services [46]. To our knowledge, this is the first LCA analysis of HISB in the regional linguistic context of Europe.

During the coronavirus pandemic in Italy, the weekly incidence rates of infections differed significantly among Italian regions [47] and were highest and vaccination rates were lowest in South Tyrol, where German and Italian speakers live together [20, 48, 49]. Experts link vaccine hesitancy in the northernmost province of Italy to a cultural disposition and historic link to Austria [21]. Confidence in accepting vaccination relies on community knowledge and trust in science, government, and public health structures [50]. Even though vaccine hesitancy was not included in the survey items, the observed linguistic effects in HISB in this study may have an impact on differences in vaccine uptake and protective behavior during the coronavirus pandemic, which remains speculative.

This study had several limitations. (i) The study database was representative of the adult population (> 18 years) living in private households and using landline phones in South Tyrol in 2014. Among vulnerable populations, smartphone ownership and language preferences impact preferences for seeking and receiving health information [12]. Smartphone ownership may have been underrepresented in this study because of design-related selection bias. Social media, which is increasingly being used for the dissemination of health-related information [51], has not been investigated. (ii) HISB from children and adolescents, which may be of particular relevance for mental health [52], was not assessed due to the inclusion criteria. (iii) The measurement of information-seeking sources was underdeveloped by insufficient differentiation between active and passive HISB. In this study, we investigated individual HISBs. There is still little data on the degree to which groups can obtain, process, understand, evaluate, and act on the information needed to make public health decisions that benefit the community [53]. Thus, the impact of this study on public health decisions in South Tyrol remains unclear. (iv) In our sample, the use of the internet as a valuable source of health-related information was low in both linguistic groups, which may have changed today, as the survey was already performed in 2014. (v) In the statistical analysis, health information indicators and the level of education were dichotomized. The response categories of health information indicators were collapsed into two categories because latent class modeling based on the original ordinal scale would have required larger sample sizes to guarantee model convergence. Similarly, we collapsed the response categories of the level of education to avoid small-cell frequencies in the multinomial logistic regression part of the model. Larger samples are required to make more fine-grained statements about health information-seeking behavior and the effects of educational status on health information-seeking patterns.

## Conclusions

Three groups of HISBs were identified in the adult South Tyrolean population:“multidimensional,” “interpersonal” and “technical/online.” In addition to age, sex, education level, and language determine group membership. Compared to the “technical/online” group, members from the “interpersonal” group were significantly more likely to have a higher age and education lower than high school, and less likely to be Italian speakers. Members of the “multidimensional” group were significantly more likely to be female, to have higher age, and be German speakers than those in the “technical/online” group. This analysis provides insights into how and where linguistic minorities in Italy obtain health information. Further research is needed to better understand how HISB can influence healthcare use, and ultimately, health outcomes. Policymakers and healthcare providers need to consider HISB for tailoring communication strategies and must provide linguistically competent health information resources to strengthen the access and use of health information sources in the digital age.

## Supplementary Information

**Additional file 1.**

**Additional file 2.**

## Data Availability

The datasets used and/or analyzed during the current study are available from the corresponding author upon reasonable request.

## Abbreviations

CI: Confidence interval
HISB(s): Health information-seeking behavior(s)
LCA: Latent class analysis
