# Supplementary material for: Health information-seeking behavior associated with linguistic group membership: latent class analysis of a population-based cross-sectional survey in Italy, August to September 2014
Source: Arch Public Health. 2022 Mar 21;80:87. doi: 10.1186/s13690-022-00847-w (PMC8935258; doi:10.1186/s13690-022-00847-w)
Supplement: Supplementary file 2 — Additional file 2. [file 13690_2022_847_MOESM2_ESM.docx]

**Additional files**

Ausserhofer et al. *Arch Public Health*

Health information-seeking behavior associated with ethno-linguistic group membership: Latent Class Analysis of a Population-Based Cross-Sectional Survey in Italy

Content

1. Measurement items of telephone survey of health information-seeking behavior

2. Survey dimensions

1. ***Measurement items of telephone survey of health information-seeking behavior***

q_01

The survey is about health issues. Do you feel ... (how) informed?

q_02

Does it occasionally happen that you ask others for advice on health issues?

q_03

How do you find out about health topics in general? I will read you a number of options, please tell me whether you use them regularly, occasionally, rarely or never.

- Articles in the newspaper or in magazines?
- TV or radio broadcasts on health issues?
- Conversations with friends or acquaintances?
- Conversations with specialists, e.g. doctors or nursing staff?
- Events or courses?
- Specialist literature, e.g. health encyclopedia or advice books?
- By chance on the Internet, e.g. if you come across it while surfing?
- On certain websites or through targeted research?
- In internet forums where you can ask or answer questions yourself?

q_04

Another question about the Internet: Where do you use it for private purposes?

q_05

Let's get back to the topic: How would you rate your current state of health?

q_06

Do you have a chronic illness or a protracted health problem?

q_07 In the past twelve months, have you been ...? How often? at your family doctor? at another doctor or at an outpatient clinic or in the hospital?

q_08

Why did you go to the doctor? Was that...?

Reasons for visiting a doctor (several options):

never went to the doctor - because of acute or specific problems - for therapy, treatment or follow-up examinations - for a routine check-up or check-up - because of something else (no information)

q_09

How about smoking If you smoke regularly or occasionally, sooner you have have you ever smoked or have you never smoked?

q_10

How often do you exercise that means that you have been moving for more than 20 minutes while doing it? out of breath or sweating?

d_11

May I ask you about your height and weight?

- Height in cm?
- Body weight in kg?

q_12

And how about stress? Do you feel very heavily burdened, fairly burdened, a little burdened or not burdened at all by demands or problems at work or in your private life?

q_13 ... I will now read you some statements and would like to know whether they apply to you: Please tell me spontaneously about each of them: applies very, fairly, a little or not at all.

- For the sake of my health, I am willing to do without things that I actually enjoy
- With minor illnesses I can rely on the self-healing powers and the immune system of my body
- Those who worry too much about their health tend to end up imagining illnesses
- I would be so embarrassed about some illnesses that I would find it difficult to confide in someone
- For a cold, traditional home remedies can be just as effective as drugs from the pharmacy
- The thought of a visit to the dentist or hospitalization makes me quite nervous
- Sometimes I don't care enough about my health due to lack of time or stress
- Sometimes you can endure slight pain instead of swallowing medication
- When I look at myself in the mirror, I am very happy with my body

q_14

There are people who prefer to go to the doctor straight away if they have health problems, and others who first see what they can do themselves. Where would you rather classify yourself? If you have health problems, go straight to the doctor - see for yourself first - depending on the situation, both

q_15

Don't go to the doctor right away: What would you do in such a case? No answer - yourself examine / treat, e.g. take a fever - ask relatives or friends - ask at the pharmacy - look up in a book - do research on the internet - wait - something else (additional mentions)

q_16

How much do you trust in health matters in general ...? Very, fairly, a little, or not at all?

- Your family physician
- The specialists in the outpatient departments or hospitals
- The pharmacists
- The advice of friends or relatives
- Information from books
- Information from the Internet
- Your own feelings or experiences

d_17

How is it with you?

- Do you always read the package insert for medication you are taking?
- Do you sometimes find the technical terminology used by doctors or on instruction leaflets difficult to understand?
- Do you have a medicine cabinet?
- Do you dare to take a temperature for yourself or others?
- Do you dare to treat smaller wounds professionally?
- Have you ever taken a first aid course?

d_18

Has it ever happened to you ...?

- Have you taken medication that you had left over, for example, without a doctor's prescription?
- Have not taken any medication that a doctor recommended to you?
- Doubted your doctor's diagnosis and consulted a second doctor to be on the safe side?
- Be so dissatisfied with your doctor that you looked for another one?

q_19 Do you actually know traditional home remedies? Yes No

q_20

Which ones do you think of spontaneously?

Herbs in general - arnica - chamomile - sage - onion - calendula - St. John's wort - other plants - honey - salts, minerals - schnapps, alcohol - vinegar, oils - cream, ointment - inhalation - aspirin, etc. - Envelopes, rubs - Teas - Homeopathy - Milk – Others

q_21

Is there anything that has changed your attitude or knowledge about health? E.g., a certain event or a particular piece of information? If so: what was that (mentions)?

- No
- Own illness
- Death / illness of loved ones
- Others

q_22

what year you were born in? (Age groups)

q_23

May I ask for the gender?

q_24

What is the language in which you think and which you speak best?

German - Italian - Ladin – Other

q_26

What is your highest school leaving certificate?

- Elementary school, no qualification
- Middle school
- Vocational training without Matura
- Matura, state high school diploma
- Master's degree
- Academic degree

d_27 / d_28 / d_29

What is your job situation? Do you have a job?

If you are employed: Do you hold a managerial position?

If self-employed: do you employ people? If yes, how many?

q_30

Employed: Do you work in a health care profession? In training: are you training in a health care profession? Other: Have you previously worked in a health care profession? Which one?

q_31

Is there a doctor or someone in your circle of friends or relatives who is involved in one Health care professional working?

q_32

How many people live in your household, including yourself?

q_32-d_37

Information on household members: Do you live with ...?

q_38

How would you describe your living environment?

q_39 Finally, I would like to ask you a very personal question. ... If you were trying to put yourself in the shoes of a dying person, what would you personally prefer? Preferred place of death:

At home - in the hospital - don't know / refused

q_40

Prefer to die at home: What do you think is most important to you?

To be in the familiar home environment

Or having family members around

Something else

q_41

Prefers to die at home and gives other reasons: What else? (mentions)

1. ***Survey dimensions***

| ***Question (q)*** | ***Dimension*** | ***Number*** |
| --- | --- | --- |
| 1, 2, 3, 4 | Extent to which informed, information behavior | 7 |
| 5, 6, 7, 8 | Subjective state of health, visits to the doctor | 4 |
| 9, 10, 11, 12 | Risk and protective factors, health behavior | 4 |
| 13 | Health attitude | 6 |
| 14, 15 | Situational information behavior | 2 |
| 16 | Trust in doctors and sources of information | 3 |
| 17, 18 | Health literacy indicators | 3 |
| 19, 20 | Familiarity of home remedies | 2 |
| 30, 31, 37, 38 | Additional socio-demographic information | 4 |
| 39, 40, 41 | Questions about the place of death | 2 |
|  | ***Total*** | 37 |
|  | Standard socio-demographics |  |
